# Experimental and Molecular Dynamics Simulation Study on Sol–Gel Conversion Process of Aluminum Carboxylate System

**DOI:** 10.3390/ma15072704

**Published:** 2022-04-06

**Authors:** Tao Luo, Yunzhu Ma, Shuwei Yao, Juan Wang, Wensheng Liu

**Affiliations:** 1 National Key Laboratory of Science and Technology on High-Strength Structural Materials, Central South University, Changsha 410083, China; 163301024@csu.edu.cn (T.L.); zhuzipm@csu.edu.cn (Y.M.); 2 Advanced Research Center, Central South University, Changsha 410083, China

**Keywords:** aluminum sol, gelation, alumina precursor fiber, molecular dynamic, rheology

## Abstract

Due to the lack of relevant in situ characterization techniques, the investigation of aluminum sol–gel progress is lacking. In this study, combined with molecular dynamics simulation and conventional experimental methods, the microstructures, rheological properties, and gelation process of the carboxylic aluminum sol system were studied. The experimental results showed that, with the increase in solid content, the microstructure of the colloid developed from a loose and porous framework to a homogeneous and compact structure. The viscosity of aluminum sol decreased significantly with the increase in temperature, and a temperature above 318 k was more conducive to improving the fluidity. The simulation results show that the increase in free volume and the connectivity of pores in colloidal framework structure were the key factors to improve fluidity. In addition, free water molecules had a higher migration rate, which could assist the rotation and rearrangement of macromolecular chains and also played an essential role in improving fluidity. The Molecular dynamics simulation (MD) results were consistent with experimental results and broaden the scope of experimental research, providing necessary theoretical guidance for enhancing the spinning properties of aluminum sol.

## 1. Introduction

The continuous alumina fiber has extensive application in aerospace, power engineering, and high-temperature thermal protection, owning to its high strength, high modulus, and superior high-temperature oxidation resistance [1,2,3,4]. The sol–gel method is considered the most effective method for preparing continuous alumina fiber, due to its accurate composition design and superior homogeneity [5,6,7]. In this method, the transition from high viscosity alumina sol to gel is necessary to obtain alumina precursor gel fiber [8,9,10]. Accurate and effective control of the colloidal composition, microstructure, and gelation transition is conducive to avoiding fatal defects such as holes, cracks, and loose structure in the subsequent sintering process [11,12,13]. However, due to the lack of in situ detection technology for quantitative characterization of an aluminum sol, research on the structural evolution and the precise control of the technological parameters in the transformation process from the high viscosity of aluminum sols to the gel fibers is lacking.

Computer simulation has been an effective method to study the solvation and gelation behaviors [14,15]. Cheng et al. revealed that the formation and fracture of bridge hydrogen bonds caused by bubbling and CO_2_ removal were the key factors leading to the CO_2_/N_2_ switchable sol–gel transition behavior [16]. Li et al. found that the increase in OH ionization degree hinders the gelation process by preventing silicic acids from approaching the nanosilica surface [17]. Huang et al. suggested that hydrophobic interactions induce methylcellulose gelation [18]. Cheng et al. studied the hydrolysis and oligomerization of Al(OC_3_H_7_)_3_ under neutral and alkaline conditions [19,20]. They found that a mixture of hydrolysis products was formed due to the decomposing of aluminum alkoxides via ligand disproportionation. Li et al. systematically studied the chemical behavior of aluminum powder with formic acid and acetic acid in neutral and acidic aqueous solutions using Density functional theory (DFT) calculation [21,22,23,24]. The formation mechanisms for the hydrolysate, dimer, and tetramer by polymerization were discussed. According to these studies, a dimer reacts with another dimer to form a tetramer through the same connection mode. However, there are few reports on the structure evolution and properties of aluminum sol in the further polymerization and gelation of the aluminum carboxylate system.

Our previous study found that the main chain length of the aluminum sol polymerization structure was limited by coordination groups [21,22,23,24]. However, hydroxyl, formic, and acetic acid have strong polarity, producing a large intermolecular force. Therefore, the microstructure and property changes in the aluminum sol–gel process can be further studied. In this paper, the microstructure, rheological properties, and gelation mechanism of carboxyl aluminum sol at different temperatures and solid content were studied by combining molecular dynamics simulation and conventional experimental methods. The morphology, functional groups, coordination structures, and rheological behavior of aluminum sols and gels were characterized. The models for aluminum colloidal with different component proportions and solid contents were constructed and analyzed based on molecular dynamics simulation. The conformation variation corresponding to the aluminum sol–gel process was analyzed by calculating the interaction energy, radial distribution function, and cohesive energy density. Furthermore, the influence mechanism of solvent content and temperature on colloidal structure and rheological properties was studied by using the free volume theory. This paper reveals the transformation mechanism of aluminum sol–gel at the molecular dynamic level, which provides an essential theoretical basis for the preparation of alumina precursor fiber.

## 2. Materials and Methods

### 2.1. Experimental Materials

**Figure 1:**
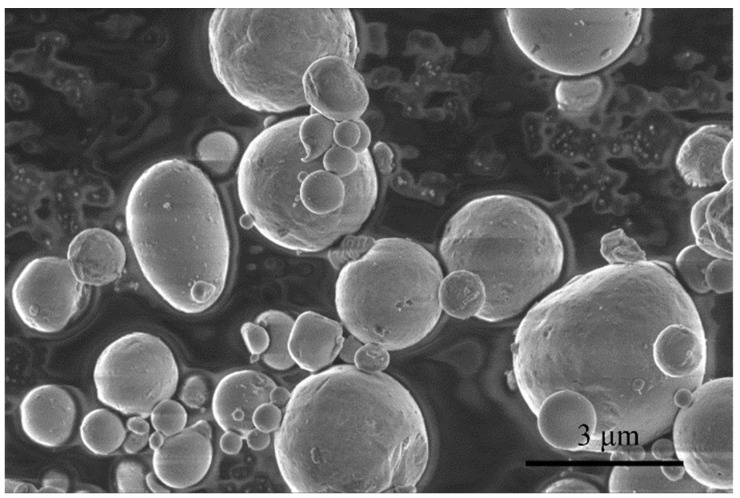
Morphology of aluminum powders.

In this study, aluminum powder (>99.5%, 1–3 μm, Aladdin Industrial Corporation, Shanghai, China) was used as the only aluminum source for preparing aluminum sols and gels. Figure 1 shows the morphology of aluminum powders. It can be seen that the diameter of aluminum powder particles is about 1–3 μm. Formic acid (99 wt%, Aladdin Industrial Corporation, Shanghai, China) and acetic acid (99.5 wt%, Aladdin Industrial Corporation, Shanghai, China) reacted with aluminum powder in an aqueous solvent (lab-made), while nitric acid (65 wt%, Sinopharm Chemical Reagent Co., Ltd., Shanghai, China) was used as pH regulator.

### 2.2. Experimental Methods

#### 2.2.1. Preparation of Aluminum Sol and Gel Fiber

The molar ratio of raw materials was Al:HCOOH:CH_3_COOH:HNO_3_:H_2_O = 1:0.69:0.50:0.31:22. First, formic acid and acetic acid were mixed with deionized water and stirred in a reactor at 368 K. Then, Al powders were added to the mixed aqueous solution for two hours. Following the addition of aluminum powder, nitric acid was slowly added to adjust the pH. The aluminum sol was obtained by continuous stirring at 368 k for 12 h. The transparent precursor sols were obtained by filtering through 1 and 0.45 μm filter membranes. Finally, the sol was concentrated under vacuum at 318 K to obtain high viscosity spinning solution. The aluminum gel fibers were prepared using a lab-made dry spinning apparatus and collected by a bobbin winder.

#### 2.2.2. Characterizations

The aluminum sol and gel samples were evenly spread in the evaporating dish and then placed in a 228 K vacuum chamber and dried for 20 h in order to avoid damaging the in situ structure of the colloidal framework during shrinkage and deformation. The skeleton morphology of the dried gel samples was obtained via scanning electron microscopy (SEM, Helios Nanolab G3 UC, FEI, Hillsboro, OR, USA).

The coordination structure of aluminum in aluminum sols and gels was measured by using ^27^Al nuclear magnetic resonance solid-state spectra (NMR, AVANCE III HD 400 M, Bruker, Fällanden, Switzerland) at a frequency of 104.2 MHz, and the chemical shift was based on 1 M Al(NO_3_)_3_·9H_2_O solution as the external reference (0 ppm).

The characteristic functional groups of aluminum sols and gels were identified using Fourier transform infrared spectroscopy (FTIR, Nicolet iS50, Thermo Fisher, Waltham, MA, USA) in the range of 400 to 4000 cm^−1^. The infrared spectrum was obtained by mixing the freeze-dried aluminum sol at 228 K with potassium bromide (KBr).

The rheological performance of aluminum sols was performed using a rotational Discovery Hybrid Rheometer (DHR-2, TA instruments, New Castle, DE, USA), equipped with a 20 mm diameter parallel plate fixture and a gap setting of 300 μm for all the measurements. The steady shear sweep and dynamic frequency sweep measurements were performed within the shear rate range between 0.1 and 100 s^−1^ and oscillation frequency range from 0.1 to 628 rad/s, respectively.

### 2.3. Computational Details

#### 2.3.1. Confirm the Aluminum Sol Component

**Figure 2:**
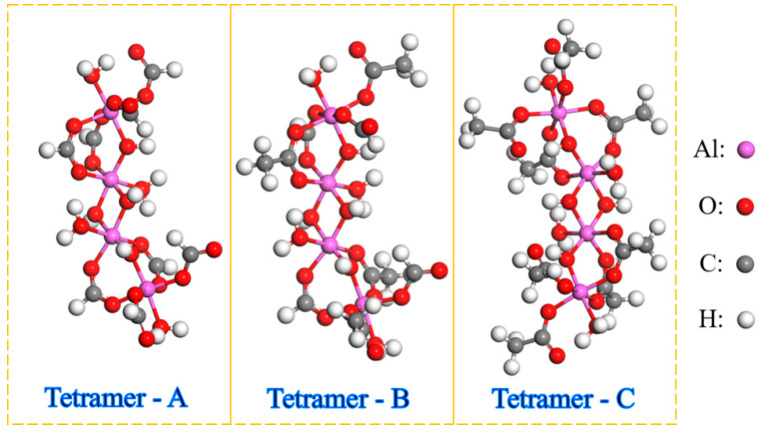
Molecular structure of three tetramers in aluminum sol.

Based on the previous studies, three kinds of tetramers with different coordination groups were formed in the Al-HCOOH-CH_3_COOH-H_2_O system [22]. The molecular structures of these three tetramers—A, B, and C—are shown in Figure 2. In molecular A, the ligands of aluminum atoms are HCOO- and OH-. In molecular B, the ligands of aluminum atoms are HCOO^−^, CH_3_COO^−^, and OH^−^. In molecular C, the ligands of aluminum atoms are CH_3_COO^−^ and OH^−^. The MD simulations were performed using Material Studio 2022 (Accelrys Inc., San Diego, CA, USA) to simulate the sol–gel conversion process. In order to ensure the effectiveness of the simulation, the proportion of oligomer components in aluminum sol should be determined first. As shown in Table 1, cubic simulation boxes containing oligomers of varying proportions were constructed with the Amorphous Cell program. Ten independent configurations were constructed for each proportion. The Forcite module was used to optimize the energy of each configuration. The molecular dynamics simulation was performed at 298 K for 2 ns in the canonical ensemble (NVT) and then at 298 K and 0.15 GPa for 2 ns in the constant-pressure, constant-temperature ensemble (NPT ). The temperature and pressure were maintained by the Nose and Souza–Martins methods, respectively. During the whole simulation process, interatomic interactions were computed by the Dreiding force field [25], and the application data of the force field are listed in Appendix A. The proportion of components with the lowest energy configuration was selected for subsequent simulation.

#### 2.3.2. Construction of Aluminum Sol Models

The lowest energy configuration was obtained by the above method, and based on the oligomer ratio of this configuration, the colloidal model of aluminum carboxylate was composed of Tetramer-A, Tetramer-B, Tetramer-C, and solvent water molecules. The molecular dynamics simulations were performed to study the atomistic models of aluminum sols with different solid content (mass fraction of alumina = 25%, 26%, 27%, 28% 29%, 30%, 31%). Ten independent configurations were constructed for each solid content system. In each configuration, the total number of tetramers (A, B, and C) was maintained at 100.

#### 2.3.3. Molecular Dynamic Simulation

The evolution of the sol structure and rheological behavior during the aluminum sol–gel process were simulated based on the above configurations. Firstly, the smart minimization method was used to optimize the energy and eliminate the local imbalance of the system, in order to obtain the lowest energy configuration. The convergence tolerance of energy was set to 0.001 kcal/mol. Then, 4 configurations representing different solid content were obtained for subsequent simulation. The molecular dynamic simulation was performed at different temperatures (258 K, 278 K, 298 K, 318 K, 338 K, 358 K) for the 4 configurations. The Group-based summation was adopted for the Coulombic interactions. The atom-based summation was applied for the van der Waals interactions, with a cutoff distance of 12.5 Å, a spline width of 0.1 Å, and a buffer width of 0.05 Å. The fractional free volume (FFV) of the system was expressed mathematically in the form of free volume/total volume. FFV values were calculated directly by the Atom Volumes and Surfaces tool in Material Studio 2022. The grid interval was 0.75 Å. The Connolly radius was 2.0 Å (referred to as the diameter of the water molecule). The mean square displacement (MSD) curves and diffusion coefficient were estimated by the Forcite analysis tools.

## 3. Results and Discussion

### 3.1. Morphology of Aluminum Sol and Gel

**Figure 3:**
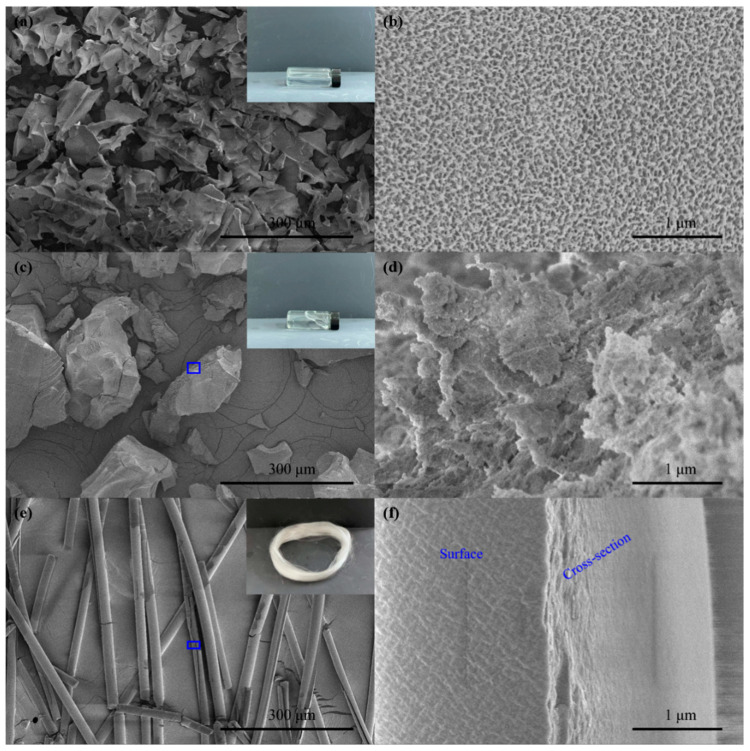
Morphologies of aluminum sols and gels with different solid contents: (**a**,**b**) 10.0 wt%; (**c**,**d**) 26.4 wt%; (**e**,**f**) 31.2 wt%.

Figure 3 shows the morphologies of three aluminum sols after freeze-drying at 228 K. The solid content of the sol was necessary to prepare the alumina precursor fiber. The aluminum sol with a solid content of 10.0 wt% was obtained after the reaction of aluminum powder with two carboxylic acids in an acidic aqueous solution. The 26.4 wt% spinnable aluminum sol with high viscosity was obtained by concentration. The 31 wt% alumina precursor fibers were obtained by spinning. As shown in Figure 3a,b, the framework of 10.0 wt% colloid was porous and spongy. The gel skeleton was easy to collapse into small blocks. The pores were caused by the volatilization of the solvent. As shown in Figure 3c,d, the cavity volume between colloidal frameworks decreased, and only nanovoids was observed in the 26.4 wt% colloid. This was because most of the solvent was removed during the concentration process. The gap between the frameworks indicated that a small amount of solvent remained between the colloidal frameworks in 26.4 wt% aluminum sol. Figure 3e,f show the uniform and dense microstructure of 31.2 wt% gel fibers, indicating that the aluminum sols had already transformed into gels during the spinning process.

### 3.2. Structural Evolution of Aluminum Sol and Gel

**Figure 4:**
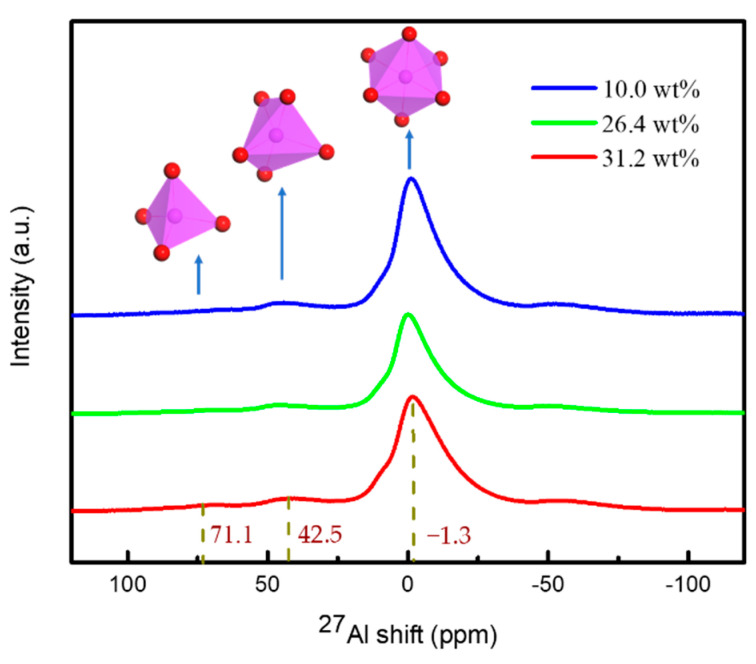
^27^Al NMR spectra of aluminum sols and gels.

The ^27^Al NMR spectra of the three colloids are shown in Figure 4 to analyze aluminum coordination structures. As evident from this figure, each spectrum had a strong peak, in the range of ±10 ppm, which is attributed to the octahedral aluminum with six coordination (AlO_6_). The peaks in the chemical shift region of five coordinated hexahedral aluminum (~42.5 ppm, AlO_5_) and four coordinated tetrahedral aluminum (~71.1 ppm, AlO_4_) were feeble, implying that the Al speciation of the three colloids was mainly AlO_6_ [26]. The results for the 26.4 wt% sample and the 31.2 wt% sample looked the same, indicating that the six coordination structure of aluminum atoms remained the same after the concentration of high viscosity aluminum sol. The removal of free water did not change the coordination state of aluminum atoms. Therefore, the increase in the solid content of aluminum sol in the concentration process had little effect on the coordination structure of aluminum atoms.

**Figure 5:**
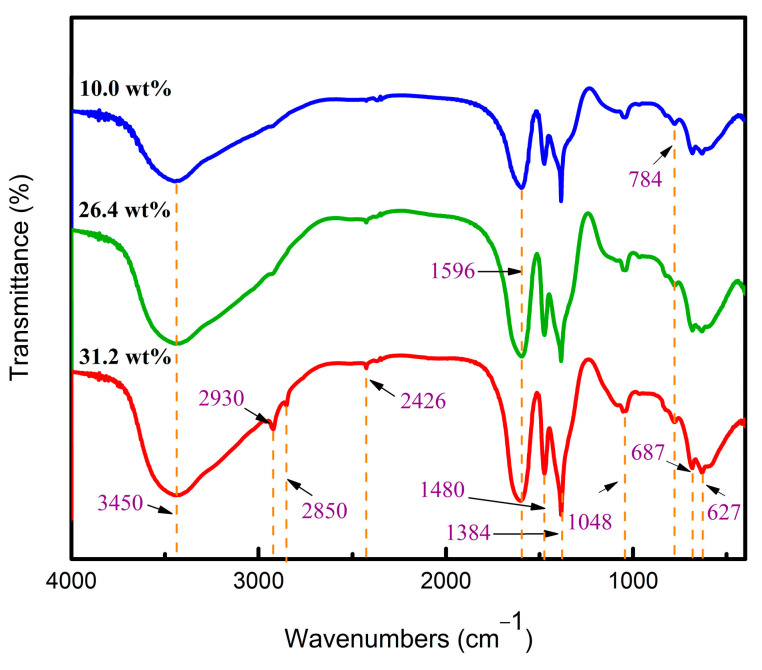
FT-IR spectra of aluminum sols and gels.

The characteristic functional groups of the three colloids were identified by FT-IR. As shown in Figure 5, the broadband at 3450 cm^−1^ corresponded to the O-H stretching vibration of hydroxyl or carboxyl groups [27,28]. The peaks at 2930 cm^−1^ and 2850 cm^−1^ were attributed to the stretching vibration of the C-H bond [27,29]. The small peak at 2426 cm^−1^ was attributed to free carboxylic acid vibrations. In addition, the three sharp peaks at 1596 cm^−1^, 1480 cm^−1^, and 1384 cm^−1^ were attributed to symmetric stretching vibrations of bridging bidentate COO^−1^ [22]. The band at 1048 cm^−1^ corresponded to Al-O-C stretching vibrations. Compared with 10 wt% aluminum sol, the 31.2 wt% gel fibers had a sharper peak at 1048 cm^−1^, indicating that oligomers might polymerize through Al-O-C connection with the increase in solid content. The broad bands around 627 and 687 cm^−1^ corresponded to the Al-O-Al bonding of aluminum oxide octahedron (AlO_6_). The intensity of the peaks around 784 cm^−1^ indicated that Al-O-Al bonding for aluminum oxygen tetrahedron (AlO_4_) was relatively low. Thus, the coordination structure of aluminum atoms was mainly alumina hexahedron (AlO_6_) for alumina sol and gel.

### 3.3. Rheological Behaviors of Aluminum Sols with High Viscosity

**Figure 6:**
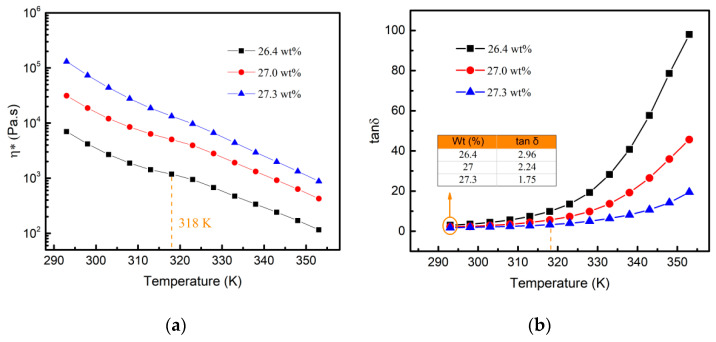
The dynamic rheological behavior of the aluminum sols at different temperatures: (**a**) complex viscosity; (**b**) tanδ.

Proper temperature and viscosity were essential to preparing aluminum gel fibers. Figure 6 shows the dynamic rheological properties of the high viscosity aluminum sol at different temperatures. The viscosities significantly increased with the increase in solid content at the same temperature. With the increase in temperature, the viscosities of the alumina sols significantly decreased.

The value of tanδ (tanδ = G″/G′) measured by small-amplitude oscillatory shear could reflect the gelation characteristics of viscoelastic specimens. The sol sample exhibited good elastic behavior, where tanδ < 1. When tanδ > 1, the increase in tanδ value corresponded to better fluidity [30,31,32]. As shown in Figure 6b, this tanδ value was increased with the rise in temperature for all sol models, indicating that the fluidity of aluminum sol improved with the increase in temperature. In addition, the tanδ value decreased with the increase in solid content, indicating that the fluidity of the system decreased. It should be noted that the viscosity of 27.3 wt% aluminum sol was very close to the gel threshold. Further increasing the solid content, the fluidity of this ultra-high viscosity sol was too poor to be used as a spinning solution.

### 3.4. Simulation of Conformational Variation in the Sol–Gel Process

Due to the lack of effective characterization methods, the conformational evolution of the colloidal structure was not yet clear in the preparation of gel fibers. In this study, the mobility of molecular chains and the interaction energy between molecules were calculated by MD simulation.

First, the proportion of different oligomers in the system should be determined. The non-bond energy and the potential energy of aluminum sol models (25 wt%) varied with oligomers component proportion, which is listed in Table 1. Negative values indicate that non-bond energy dominates the attraction between solvent molecules and oligomers [33,34]. The results demonstrated that the non-bond energy of aluminum sol models decreased with the decrease in B proportion. It has been reported that pure aluminum formoacetate is one of the best raw materials for preparing alumina fiber. Therefore, the ratio of n(A):n(B):n(C) = 4:15:1 was used to simulate the conformational evolution of the sol–gel process.

**Figure 7:**
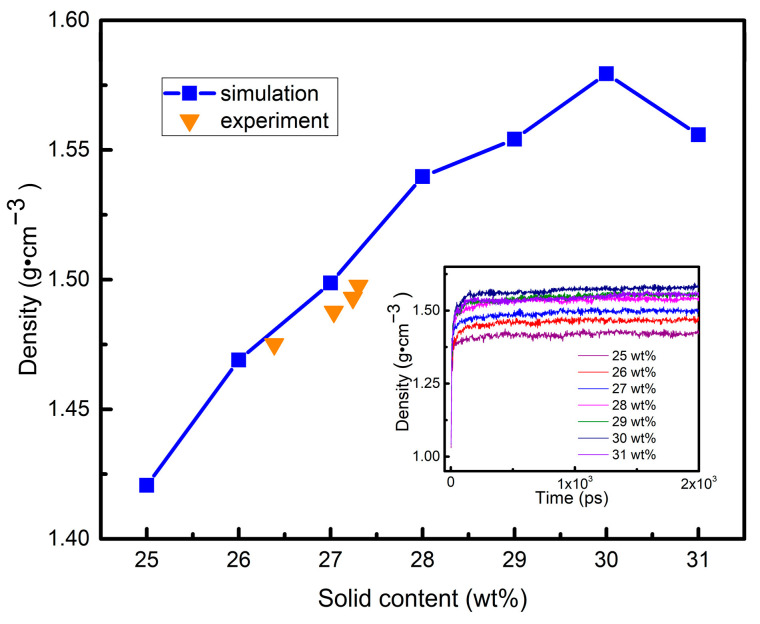
Calculation and experimental density of aluminum sol with different amounts of solid content at 298 k.

The structural transformation of high-viscosity aluminum sol is more important to studying the gelation process. Thus, this paper simulated the high-viscosity aluminum sol. Based on the ratio of n(A):n(B):n(C) = 4:15:1, several calculation models were constructed in combination with the variation interval of solid content (25 wt%~31 wt%) during spinning. High viscosity aluminum sol (25 wt%) was squeezed into the air through spinnerets. The solvent of the primary fiber was volatilized during the continuous stretching deformation process to obtain the gel fiber (31 wt%). Therefore, the models of different solid content were established by adjusting the number of water molecules. The theoretical and experimental densities were compared to verify the validity of these models [35]. Figure 7 illustrates the calculation and experimental density of the aluminum carboxylate system with different amounts of solid content at 298 k. As can be seen, the measured densities of high viscosity aluminum sols were very close to the calculated densities. In addition, with the increase in solid content, the increasing trend and amplitude of theoretical and measured density were the same. Therefore, these models were reliable and could be used in the next step.

**Figure 8:**
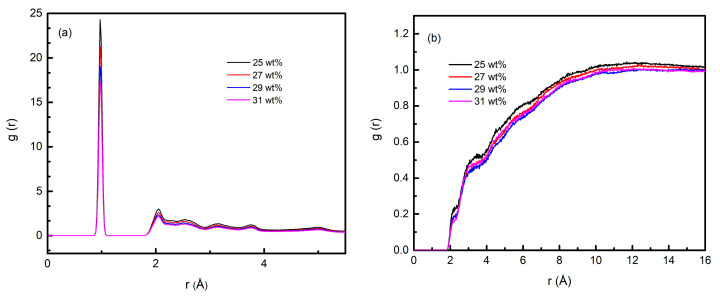
The g(r) of inter (**a**)/intra (**b**) molecular oxygen atoms (B-O) and hydrogen atoms (B-H) of Tetramer-B in the models with different solid contents.

Radial distribution function (RDF) g(r) was first calculated to reflect the microstructure of the aluminum carboxylate system. Figure 8 shows the g(r) of intra/intermolecular oxygen atoms (B-O) and hydrogen atoms (B-H) of Tetramer-B in the system with different amounts of solid content since Tetramer-B was the main polymerization structure. Inter-g(r) could provide information about the order of polymerization structure. As shown in Figure 8a, the characteristic peaks of the system mainly appear at 0.97 Å, 2.04 Å, 2.55 Å, 3.15 Å, and 3.78 Å. These peaks illustrated the bond connectivity of the polymerization chain. The characteristic peaks at 0.97 Å and 2.04 Å indicated the amorphous microstructure of the system. Intra-g(r) revealed the mode and essence of intermolecular interaction. As shown in Figure 8b, there were two platform regions in the range of 2.1 Å~2.5 Å and 3 Å~4 Å, respectively corresponding to the two intermolecular interaction modes of hydrogen bond and van der Waals force in the system. In addition, the distance between some B-O … B-H pairs was less than 2.1 Å, representing a stronger intermolecular force, which might be conducive to further polymerization between oligomers.

**Figure 9:**
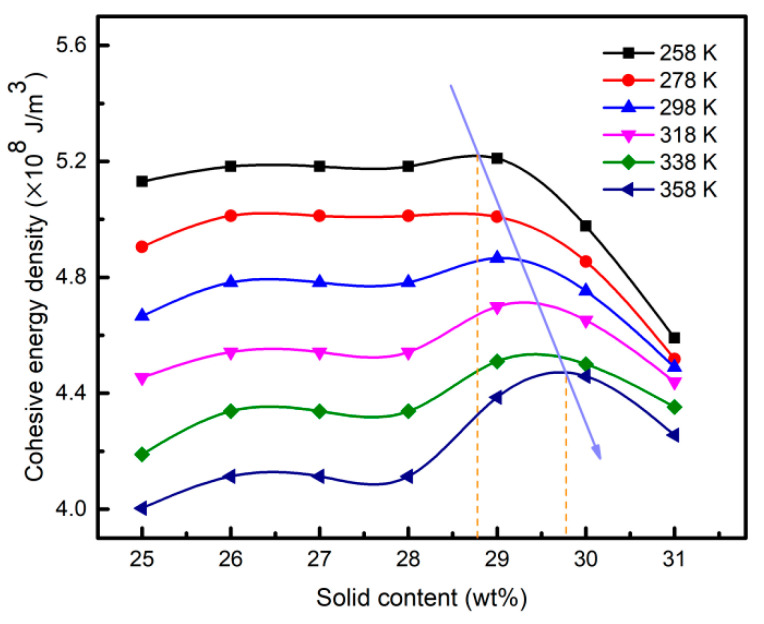
Cohesive energy density of aluminum sols with different amounts of solid content.

The cohesive energy density (CED) was a parameter that depends on breaking all intermolecular interactions in a unit volume [34]. It was reported that the maximum value of CED reflected the maximum intermolecular interaction in the unit volume, corresponding to the gel transition point of the system. As shown in Figure 9, the CED decreased with the increase in temperature, and the increase rate of solid content corresponding to its maximum value was about 1%, meaning that the solid content of the gel transition point increased slightly with the increase in temperature. Thus, it was essential to achieve precise control of the technological parameters during the curing process in order to obtain continuous uniform gel fibers. In the above study, the changing trend of temperature and solid content on rheology was consistent with the dynamic rheological test. Therefore, intermolecular interaction was one of the critical factors that influenced the gelation of aluminum sol.

### 3.5. Free Volume Characteristics of Aluminum Sols and Gels

**Figure 10:**
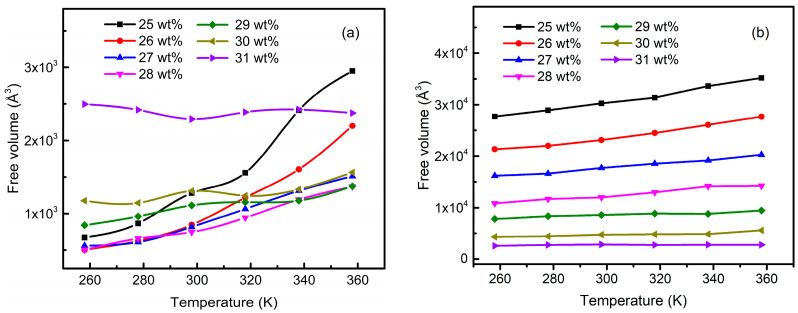
Free volume of aluminum sols with different solid contents: (**a**) with solvents; (**b**) without solvents.

**Figure 11:**
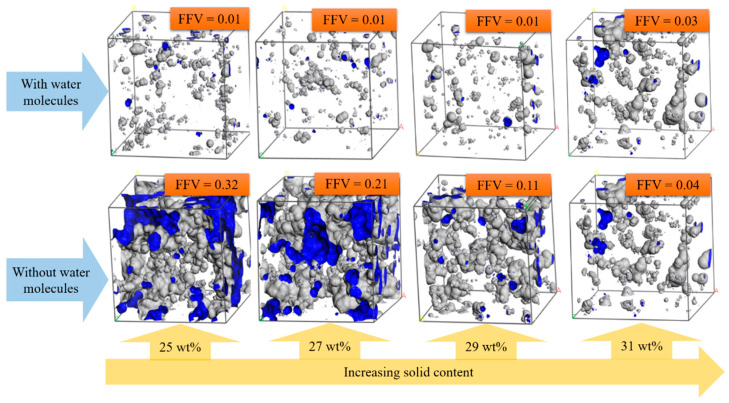
Free-volume snapshot of aluminum sols with different amounts of solid content at 298 K.

Rheological variation is an essential characteristic of the sol–gel process. The free volume theory has been used to successfully describe the polymeric phenomena, such as T_g_ and η [36]. Free volume is the available space, which controls the liquidity by restricting/allowing the molecular movements. Figure 10 shows the free volume of aluminum sols with different amounts of solid content. From Figure 10a, it can be inferred that the free volume increased with the increase in temperature, indicating that the molecular chain could be rotated and rearranged in a larger space with the temperature increase. The increase in free volume resulted in improved liquidity. However, the change in free volume tended to be gentle with the increase in temperature when the solid content was above 29 wt%. Thus, the rheological properties of the systems were no longer changed. In other words, the sols with solid content above 29 wt% began to solidify into gels. Although the free volume of 31 wt% was the largest, this did not mean that the rheological properties of the systems were improved, especially considering the reduced density of the systems. These phenomena were related to the volatilization of water molecular solvents, as shown in Figure 10b and Figure 11.

Figure 10b illustrates the free volume after deleting the solvent water molecules, and the value sharply decreased with the increase in solid content. Figure 11 shows snapshots of the free volume before and after deleting the water molecules in different solid content systems at 298 K. The parts surrounded by the gray area are the free volume. As can be seen from Figure 11, when the model contained water molecules, FFV did not change with the increase in solid content but increased slightly at 31 wt%. However, FFV decreased significantly with the increase in solid content when there were no water molecules in the model. Especially in the range of 25 wt%~29 wt%, there were plenty of connected voids conducive to the movement of molecular chains. In addition, the density of the system decreased because the total volume of the gel did not change considerably after the gelation near 30 wt% (as shown in Table 2). However, the water solvent still volatilized from the gel skeleton and left vacancies, resulting in an increase in free volume. In contrast, after deleting the water molecules, the results could better reflect the law that the fluidity of the system deteriorates with the increase in solid content. Thus, the water solvent content is a dominant factor in the gel transition in the sol–gel process. The reason might be that, compared with the polymerized molecular chain, the volume of water molecules was minimal, and the higher diffusion rate made it easy to move in the colloidal skeleton and assisted the macromolecular chain in rotation and rearrangement.

### 3.6. Component Migration Characteristics in the Sol–Gel Process

**Figure 12:**
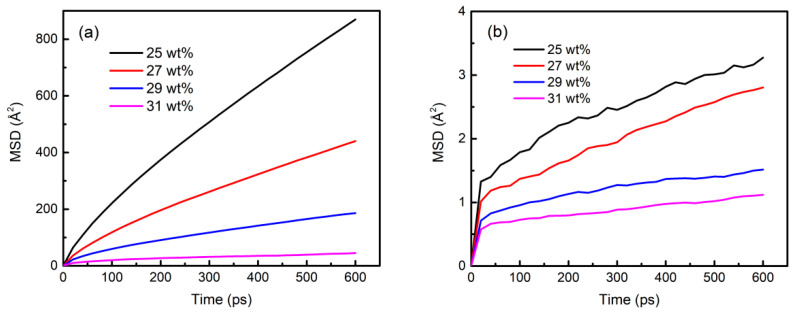

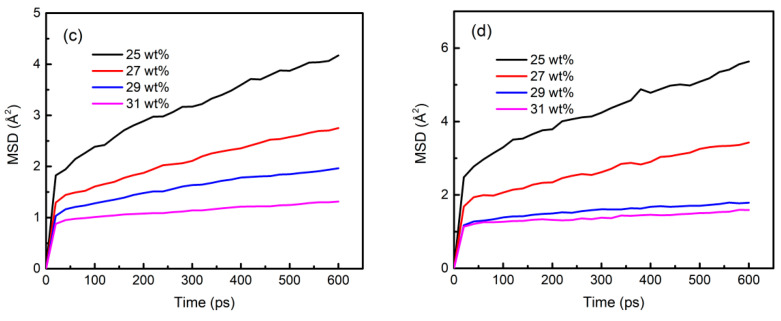
Mean squared displacement (MSD) curves of different components in aluminum sols at 298 K: (**a**) water molecular; (**b**) A; (**c**) B; (**d**) C.

The slope of MSD curves was used to characterize the mobility of components [34] in aluminum sol models, and the variation in MSD curves of chains with different amounts of solid content at 298 K was compared in Figure 12. It could be seen that the slope of MSD curves decreased with decreasing the solvent content, illustrating that the volatilization of the solvent limited the mobility of the components, including solvent water molecules. The relevant diffusion coefficients are listed in Table 3. The diffusion coefficients of each component decreased with the increase in solid content, and the value of water molecules was much higher than that of Tetramer-A, -B, and -C. The movement mode of molecules could be speculated by comparing the diffusion coefficients and molecular radius of components. Since the diameter of water molecules was about 2 Å, the motion mode of water molecules might be rotation and translation in the range of low solid content (<29 wt%), and the polymerization chains might move through micro-region rotation due to their larger molecular diameter. In addition, the main chain structure dominated by octahedral aluminum (AlO_6_) might affect the flexibility of macromolecules. Therefore, to improve the fluidity of the system, it was necessary for water molecules to improve the movement of macromolecular chains in the connected pores within a colloidal framework. When the solid content increased to 31 wt%, the water molecules were challenging to move in the closed pores of the colloidal framework, and the colloid completely lost its fluidity, which was a typical gel characteristic. Thus, the aluminum sol was completely transformed into the gel.

## 4. Conclusions

In the present investigation, a combined computational and experimental study was used to evaluate the evolution of microstructures, rheological properties, and gelation during the sol–gel process. The experimental results showed that with the increase in solid content, the microstructure of the colloid developed from a loose and porous framework to a homogeneous and dense structure. The Al speciation of aluminum sol and gel was mainly aluminum oxide hexahedron (AlO_6_). The viscosity of high viscosity aluminum sol decreased greatly with the increase in temperature, and the temperature above 318 k might be more conducive to improving the fluidity. The simulation results show that the increasing range of the solid content of the gel transition point was about 1% in the range of 258 K~358 K, which played an essential role in guiding precise control of the technological parameters in the curing process to obtain continuous uniform gel fibers. Further analysis of microstructure evolution showed that the increase in free volume and the connectivity of pores in colloidal framework structure were the key factors to improve fluidity. In addition, free water molecules had a higher migration rate, assisting the rotation and rearrangement of macromolecular chains. The movement ability and space of free water molecules in the colloidal framework were the core reasons for the gelation evolution of high viscosity aluminum sol. The MD results are consistent with experimental results and broaden the scope of experimental research, playing an essential role in determining the structure and properties of the final materials.

## Figures and Tables

**Table 1:** The non-bond energy and the potential energy of aluminum sol models varied with component proportion.

A:B:C	E_Non-Bond_ /kcal∙mol^−1^	△E /kcal∙mol^−1^	E_po_ /kcal∙mol^−1^	△E /kcal∙mol^−1^
4:15:1	−3752.0	0.0	69,112.5	0.0
5:13:2	−3695.0	57.1	69,211.1	98.6
6:11:3	−3658.3	93.7	69,259.5	147.0
7:9:4	−3641.7	110.3	69,289.3	176.8
8:7:5	−3630.9	121.1	69,318.9	206.4
9:5:6	−3602.0	150.1	69,374.0	261.5
10:3:7	−3584.6	167.4	69,424.3	311.8
11:1:8	−3584.2	167.8	69,437.5	325.0

**Table 2:** Volume parameters of aluminum sol systems at 298 K.

	Cell Dimension (x×y×z) (Å^3^)	Cell Volume (Å^3^)	W Volume (Å^3^)
25.0 wt%	50.92 × 45.50 × 42.17	94,851.285	28,997.936
26.0 wt%	46.71 × 40.38 × 46.79	88,147.821	22,257.313
27.0 wt%	43.88 × 41.42 × 45.97	83,540.026	16,893.963
28.0 wt%	40.96 × 43.91 × 45.20	78,364.597	11,247.907
29.0 wt%	39.59 × 45.32 × 43.52	75,342.807	7470.678
30.0 wt%	36.89 × 45.35 × 43.57	71,531.849	3437.288
31.0 wt%	43.16 × 39.97 × 41.09	70,466.882	270.524

**Table 3:** Diffusion coefficients of different components in aluminum sol at 298 K.

	Diffusion Coefficient/Å^2^∙ps^−1^			
	W	A	B	C
25.0 wt%	0.20086	0.000475	0.000549	0.000698
27.0 wt%	0.09936	0.000468	0.000342	0.000436
29.0 wt%	0.03860	0.000128	0.000173	0.000108
31.0 wt%	0.00735	0.000124	0.000096	0.000102

## Data Availability

Not applicable.

## Appendix A. DREIDING Parameters

**Table A1:** Force field type used in molecular dynamics simulation of aluminum sol.

Element Symbol	Type	Description	Hybridization
Al	Al3	Aluminum	Tetrahedral
C	C_3	Carbon, sp3	Tetrahedral
C	C_R	Carbon, aromatic	Trigonal
H	H_	Hydrogen	No hybridization
H	H___A	Hydrogen, involved in hydrogen bonds	No hybridization
O	O_2	Oxygen, sp2	Trigonal
O	O_3	Oxygen, sp3	Tetrahedral
O	O_R	Oxygen, aromatic	Trigonal

## References

[1] Ruggles-Wrenn M.B., Ozer M. (2010). Creep behavior of Nextel^TM^ 720/alumina-mullite ceramic composite with ±45° fiber orientation at 1200 °C. Mater. Sci. Eng. A.

[2] Tariq M., Hanif F., Ashraf A. (2011). Overview of Nextel TM based structures for Space applications. J. Space Technol..

[3] Jiang R., Sun X., Liu H., Liu Y., Mao W. (2021). Microstructure and mechanical properties improvement of the Nextel^TM^ 610 fiber reinforced alumina composite. J. Eur. Ceram. Soc..

[4] Guel N., Hamam Z., Godin N., Reynaud P., Caty O., Bouillon F., Paillassa A. (2020). Data merging of ae sensors with different frequency resolution for the detection and identification of damage in oxide-based ceramic matrix composites. Materials.

[5] Rutkowska I., Marchewka J., Jeleń P., Odziomek M., Korpyś M., Paczkowska J., Sitarz M. (2021). Chemical and structural characterization of amorphous and crystalline alumina obtained by alternative sol-gel preparation routes. Materials.

[6] Zhang H., Hang Y., Qin Y., Yang J., Wang B. (2014). Synthesis and characterization of sol-gel derived continuous spinning alumina based fibers with silica nano-powders. J. Eur. Ceram. Soc..

[7] Chen X., Gu L. (2009). Spinnablity and structure characterization of mullite fibers via sol-gel-ceramic route. J. Non-Cryst. Solids.

[8] Ádám P., Temesi O., Dankházi Z., Voniatis C., Rohonczy J., Sinkó K. (2021). Various colloid systems for drawing of aluminum oxide fibers. Ceram. Int..

[9] Chen X., Gu L. (2009). Structural evolution of sol-gel derived mullite fibers with different solid contents during sintering. J. Mater. Process. Technol..

[10] Cheng M., Liu W., Yao S., Wang J., Ma Y. (2021). Comparing the phase transformation of continuous alumina fiber and xerogels derived from the same precursor. J. Sol-Gel Sci. Technol..

[11] Duan N., Zhang X., Lu C., Zhang Y., Li C., Xiong J. (2022). Effect of rheological properties of AlOOH sol on the preparation of Al_2_O_3_ nanofiltration membrane by sol-gel method. Ceram. Int..

[12] Liu L., Wang J., Ma Y., Liu W., Yao S. (2020). Preparation of continuous alumina fiber with nano grains by the addition of iron sol. Materials.

[13] Liang C., Liu W., Liu Q., Gao Y., Liu J., Wang J., Yao S., Ma Y. (2021). The formation of core-sheath structure and its effects on thermal decomposition and crystallization of alumina fibers. Ceram. Int..

[14] Nie Y., Zhan H., Kou L., Gu Y. (2021). Atomistic Insights on the Rheological Property of Polycaprolactone Composites with the Addition of Graphene. Adv. Mater. Technol..

[15] Yu D., Mao G., Cai H., Wang S., Liu J., Pan P., Bao Y. (2021). Free volume characteristics of 2,2-bistrifluoromethyl-4,5-difluoro-1,3-dioxole-co-tetrafluoroethylene copolymers: Effect of composition and molecular weight. J. Polym. Sci..

[16] Yuan C., Chen D.J., Ye Q.X., Xiao K., Hao L.S., Nan Y.Q. (2021). CO_2_/N_2_-switchable sol–gel transition based on NaDC/NaCl solution: Experiments and molecular dynamics simulations. J. Mol. Liq..

[17] Wen L., Xu J., Yang Q., Zhang F., Li F., Zhang L. (2020). Gelation process of nanosilica sol and its mechanism: Molecular dynamics simulation. Chem. Eng. Sci..

[18] Huang W., Dalal I.S., Larson R.G. (2014). Analysis of solvation and gelation behavior of methylcellulose using atomistic molecular dynamics simulations. J. Phys. Chem. B.

[19] Cheng X., Liu Y., Chen D. (2011). Mechanisms of Hydrolysis–Oligomerization of Aluminum Alkoxide Al(OC_3_H_7_) 3. J. Phys. Chem. A.

[20] Cheng X., Ding W., Liu Y., Chen D. (2013). Mechanistic investigations of Al(OH)3 oligomerization mechanisms. J. Mol. Model..

[21] Li C., Liu W., Luo T., Cheng M., Liu Q., Wang J., Yao S., Ma Y. (2021). Effect of formic-acid-to-acetic-acid ratio on the structure and spinnability of aqueous aluminium sol of alumina fibre. Ceram. Int..

[22] Li C., Liu W., Wang J., Yao S., Ma Y. (2021). A density functional theory study on the structure formation of Al(III) carboxylate complexes in aqueous aluminum sols. Int. J. Quantum Chem..

[23] Li C., Liu W., Ma Y. (2019). DFT Studies on the Al-Speciation and Its Structure in Aqueous Aluminum Sol Formed by Aluminum Formoacetate. J. Phys. Chem. B.

[24] Li C., Liu W., Ma Y. (2019). Influence of H_3_O+ on the structure formation of oligomers in aluminium sols prepared from basic aluminium acetate: Experiments and computations. J. Mol. Liq..

[25] Boyd P.G., Moosavi S.M., Witman M., Smit B. (2017). Force-Field Prediction of Materials Properties in Metal-Organic Frameworks. J. Phys. Chem. Lett..

[26] Haouas M., Taulelle F., Martineau C. (2016). Recent advances in application of 27Al NMR spectroscopy to materials science. Prog. Nucl. Magn. Reson. Spectrosc..

[27] Liu L., Cai W., Dang C., Han B., Chen Y., Yi R., Fan J., Zhou J., Wei J. (2020). One-step vapor-phase assisted hydrothermal synthesis of functionalized carbons: Effects of surface groups on their physicochemical properties and adsorption performance for Cr(VI). Appl. Surf. Sci..

[28] Persson P., Karlsson M., Öhman L.O. (1998). Coordination of acetate to Al(III) in aqueous solution and at the water-aluminum hydroxide interface: A potentiometric and attenuated total reflectance FTIR study. Geochim. Cosmochim. Acta.

[29] Ghaderi S., Ramazani S.A., Haddadi S.A. (2019). Applications of highly salt and highly temperature resistance terpolymer of acrylamide/styrene/maleic anhydride monomers as a rheological modifier: Rheological and corrosion protection properties studies. J. Mol. Liq..

[30] Mills J.N., Wagner N.J., Mondal P. (2021). Relating chemical composition, structure, and rheology in alkali-activated aluminosilicate gels. J. Am. Ceram. Soc..

[31] Lakkegowda Y., Ammannappa R., Ananthamurthy S. (2014). Investigations on rheological properties and gelation of tasar regenerated silk fibroin solution. J. Appl. Polym. Sci..

[32] Derkach S.R., Ilyin S.O., Maklakova A.A., Kulichikhin V.G., Malkin A.Y. (2015). The rheology of gelatin hydrogels modified by κ-carrageenan. Lwt.

[33] Gan Y., Cheng Q., Wang Z., Yang J., Sun W., Liu Y. (2019). Molecular dynamics simulation of the microscopic mechanisms of the dissolution, diffusion and aggregation processes for waxy crystals in crude oil mixtures. J. Pet. Sci. Eng..

[34] Lin D., Li R., Li T., Zi Y., Qi S., Wu D. (2021). Effects of pre-imidization on rheological behaviors of polyamic acid solution and thermal mechanical properties of polyimide film: An experiment and molecular dynamics simulation. J. Mater. Sci..

[35] Lin L., Kedzierski M.A. (2020). Density and viscosity of a polyol ester lubricant: Measurement and molecular dynamics simulation. Int. J. Refrig..

[36] Sethi S.K., Manik G. (2021). A combined theoretical and experimental investigation on the wettability of MWCNT filled PVAc-g-PDMS easy-clean coating. Prog. Org. Coat..

